#  "Prostate Cancer Proteomics" Database 

**Published:** 2010

**Authors:** S.S. Shishkin, L.I. Kovalyov, M.A. Kovalyova, K.V. Lisitskaya, L.S. Eremina, A.V. Ivanov, E.V. Gerasimov, E.G. Sadykhov, N.Y. Ulasova, O.S. Sokolova, I.Y. Toropygin, V.O. Popov

**Affiliations:** Bach Institute of Biochemistry, Russian Academy of Sciences; Lomonosov Moscow State University; Orekhovich Institute of Biomedical Chemistry, Russian Academy of Medical Sciences

**Keywords:** proteomics, prostate cancer, digital database

## Abstract

A database of Prostate Cancer Proteomics has been created by using the results of a proteomic study of human prostate carcinoma and benign hyperplasia tissues, and of some human-cultured cell lines (PCP, http://ef.inbi.ras.ru). PCP consists of 7 interrelated modules, each containing four levels of proteomic and biomedical data on the proteins in corresponding tissues or cells. The first data level, onto which each module is based, is a 2DE proteomic reference map where proteins separated by 2D electrophoresis, and subsequently identified by mass-spectrometry, are marked. The results of proteomic experiments form the second data level. The third level contains protein data from published articles and existing databases. The fourth level is formed with direct Internet links to the information on corresponding proteins in the NCBI and UniProt databases. PCP contains data on 359 proteins in total, including 17 potential biomarkers of prostate cancer, particularly AGR2, annexins, S100 proteins, PRO2675, and PRO2044. The database will be useful in a wide range of applications, including studies of molecular mechanisms of the aetiology and pathogenesis of prostate diseases, finding new diagnostic markers, etc.

##  Introduction 

The first decade of the post-genome era was marked by a rapid development in the field of bioinformatics, the extension of major databases (such as NCBI and UniProt), and the creation of specialised information resources for biomedical research in many countries [1-4]. The impressive resources created in Ireland (UCD-2DPAGE, http://proteomics-portal.ucd.ie:8082/cgi-bin/2d/2d.cgi) [2] and India (Human Proteinpedia, www.humanproteinpedia.org) [3] make the state of things in Russia pale in comparison.

Currently, one of the most important tasks for bio-medical research is to find efficient prostate cancer (PCa) biomarkers which would enable new diagnostic methods [5-8]. The fact that in recent years the PCa incidence rate has dramatically increased worldwide [9, 10], and particularly in Russia, making PCa the most frequent male oncological disease in some countries [11, 12], is reason enough to pay close attention to this disease. In early diagnostics of PCa at the moment it is important to establish the presence of one of the most studied biomarkers, the so-called Prostate-Specific Antigens (PSA), in the blood. The test, however, is known to produce a significant number of false-positive and false-negative results, leading to the wrong clinical and financial outcomes [5, 7]. Therefore, in the U.S. and in other Western countries, new PCa biomarkers are being actively sought, an initiative recently stimulated by the development of proteomic and other post-genome technologies [6, 8, 13].

Since 2005, the Bach Institute of Biochemistry, in collaboration with other research and medical institutions, has been researching new PCa biomarkers by utilising various proteomic technologies [14, 15]. In 2009, the "Prostate Cancer Proteomics" (PCP, http://ef.inbi.ras.ru/) national database was created in order to facilitate this research, summarising experimental and referenced published data and providing links to several other biomedical Internet databases. This paper describes the structure and capabilities of the new, extended PCP version.

##  Materials and methods 

**Biomaterials**

- Biopsy and surgical samples of prostate tissues from patients with PCa ( *n * = 72) and benign prostatic hyperplasia (BPH, *n * = 69) were provided by staff members of the Urology Department of the Botkin Clinical Hospital (Moscow). Diagnosis was performed using clinical, histological, and immunochemical (PSA level) tests. Histological verification was performed via U.S.-controlled transrectal multifocal needle biopsy; up to 18 tissue samples from various prostate zones per patient were taken [16, 17]. All PCa cases were found to be adenocarcinoma. Gleason score was determined by following the standard procedure [16, 17].

In parallel tests, we analysed the proteins of the PC-3 (ACC 465, DU-145 (ACC 261), and BPH-1 (ACC 143) cell cultures purchased from the German Collection of Microorganisms and Cell Cultures, as well as the proteins of cultured cells of the LNCaP line provided by Dr. I. G. Shemyakin (Obolensk National Science Centre for Applied Microbiology and Biotechnology). The cells were cultured in the RPMI-1640 medium with HEPES, sodium pyruvate, gentamicine and 20% fetal bovine serum (FBS) [18], using cell culture plastic (Costar, USA and Nunc, Denmark) in a CO_2_ -incubator (Sanyo, Japan). In addition, we studied proteins from the cultured cells of two lines of human rhabdomyosarcoma (A-204 and RD) purchased from the Ivanovsky Virology Institute, RAMS, and proteins from the cultured normal human myoblasts kindly provided by Dr T. B. Krohina [19].

**Fig. 1:**
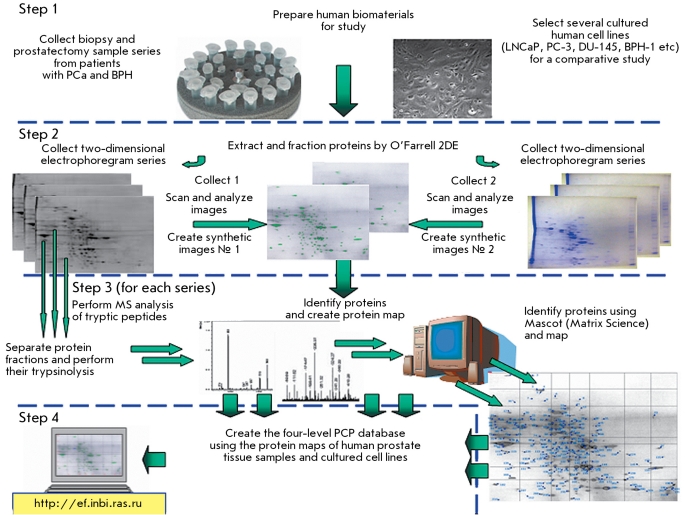
Main steps in the proteomic study of prostate tissue samples from patients with malignant and benign tumors, as well as from several cultured human cell lines.

The preparation of protein extracts, their O'Farrell 2DE fractioning, Coomassie Blue R-250 and silver nitrate staining, and 2DE analysis were performed following the techniques described in [20, 21]. In addition, we used a 2DE procedure with isoelectric focusing using IPG-PAGE and Ettan IPGphor 3 kit (GE Healthcare), according to the manufacturer's protocol. Proteins were identified with MALDI-TOF MS and MS/MS using an Ultraflex instrument (Bruker) at a 336-nm UV laser beam in a 500-8000 Da cation mode calibrated using reference trypsin autolysis peaks and processed with Mascot software, Peptide Fingerprint option (Matrix Science, USA) [21, 22]. The proteins were identified by matching experimental masses with the masses of proteins listed in the NCBI Protein and SwissProt/TrEMBL databases. The accuracy of monoisotopic masses measured in the reflection mode calibrated with autolytic trypsin peaks was 0.005%, and the accuracy of the fragment masses was ±1 Da. Hypothetical proteins identified with MALDI-TOF MS corresponding to fragments of the full-size proteins, which are products of corresponding genes, were revealed with MS/MS. The molecular masses of protein fractions were determined using the ultrapure recombinant protein sets SM0661 (10-200 kDa) and SM0671 (10-170 kDa) (Fermentas). The measurement of the optical density of 2DE images and/or their fragments was performed following scanning (Epson expression 1680) or digital photography (Nikon 2500 or Canon PowerShot A1000 IS). Digital image processing with densitometry of the protein fractions was performed with Melanie ImageMaster, versions 6 and 7 (Genebio).

**Fig. 2:**
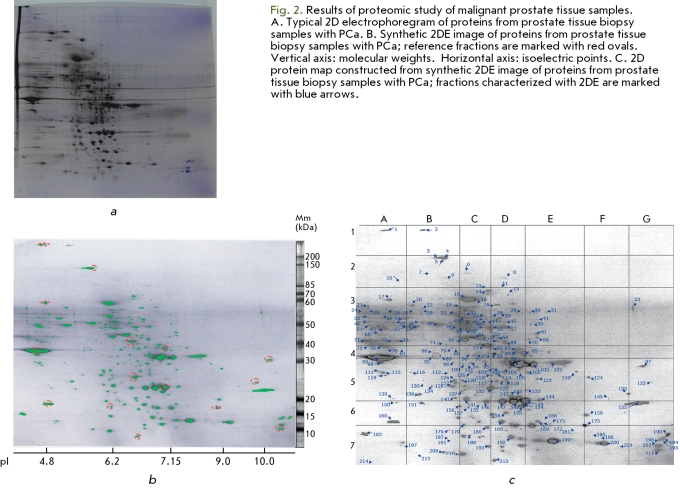


 Data logging and processing for the Prostate Cancer Proteomics multilevel database were done with various software packages, including MapThis!, Molly Penguin Software, Mozilla Firefox, and some Microsoft Office applications. A MySQL-based interactive database was used which could be updated and modified online using any computer with Internet connection. The BIOSTAT and Microsoft Office Excel 2003 software packages were used for statistical analysis. 

##  Results and discussion 

According to the conventional proteomics strategy developed in the late 20 ^th^ century, the national PCP database was created in several consecutive steps which involved systematic characterisation of proteins in prostate tissue samples obtained from benign and malignant tumors (Fig. 1) ([23, 24]). Proteins from several cultured human cell lines were studied in parallel experiments (Fig. 1).

 The first step was to make series of 2DE protein samples (50 or more) by fractioning dozens of bioptates or prostate tissue samples (from 30 or more patients). Figure 2A illustrates a typical 2DE of the PCa prostate tissue proteins. The 2DE series for cell line proteins were created with 20 2DE, taking into account the homogeneity of the analyte. 

**Fig. 3:**
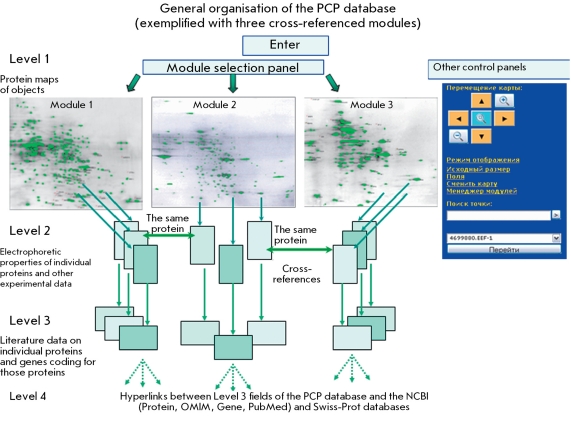
General organisation of the "Prostate Cancer Proteomics" (PCP) database.

The distribution of 2DE protein fractions was registered and stored as graphic *.tif files. The images of the entire 2DE and (in some cases) their segments were produced by scanning and/or digital photography. The relevance of the selected 2DE was assessed by comparing protein fractioning results using digital image matching [23, 24].

The second step was to construct synthetic 2D maps of the proteins. 2DE images from each series were standardised with Melanie ImageMaster software using 15 selected reference points corresponding to easily identifiable major protein fractions. Fig. 2B shows reference points on the 2DE images of proteins from prostate tissue samples.

Each image was analysed using the Cummings technique [25] with some modifications [20, 24]. The analysis was based on dividing the images into 49 rectangular fragments, the sides of which formed six horizontal and six vertical standard lines and the sides of the 2DE image itself. The points for plotting the horizontal lines were determined by special molecular-weight-marker proteins, which were placed on each gel plate before fractioning in the second direction (SDS-PAGE). Thus, the protein fractions located on the corresponding horizontal lines have identical molecular weights. For plotting the vertical lines, protein markers with previously measured pI values were used [20, 24]. As a result, each image analyzed consisted of 49 rectangular fragments, each usually containing no more than 10 protein fractions (only 4 fragments contained more than 20 fractions). Image fragmenting significantly simplified the image comparison and construction of synthetic 2D maps.

 The described procedure was performed with the 60 best 2DE images of proteins from BHP samples and with 70 2DE images of proteins from PCa samples. The comparison of standardised BHP and PCa 2DE images showed that the coordinates of more than 95% of protein fractions were constant. Quantitative or qualitative variations in the coordinates were observed for less than 5% of the fractions. The variations could be caused by genetic factors (e.g. single nucleotide polymorphism) or a different expression of corresponding genes, as well as differences in tissue composition of the samples and the pathology's intensity. 

Having made sure that the positions of the majority of the protein fractions were constant, we were able to construct the 2D maps of prostate tissue proteins from patients with BHP and PCa. After that, we performed a fragment-by-fragment comparison of the 2D maps constructed. The fraction patterns in BHP and PCa maps were found to be quite similar, the difference being that about 20 fractions present in the PCa map were either present in a much smaller amount in the BHP map or absent altogether. We paid particular attention to those fractions in further study, as described below. In general, as a result of our analysis, an integrated synthetic 2D map of human prostate proteins was constructed that contains more than 200 protein fractions in the ranges of Mw 8.5-450 kDa and pI 4.5-11.5 (Fig. 2C). Each fraction was assigned a unique seven-digit number; the first four digits representing the logarithm of the fraction's molecular weight, and the next three digits representing the value of the isoelectric point expressed in units described in [20, 24].

**Table 1:** Description of the PCP modules and the number of identified proteins by modules.

Modules-synthetic maps of proteins in specific objects (fractioning technique)	Proteins identified
Prostate biopsy samples, PCa and BPH (IEF-PAGE*)	165
LNCaP (IEF-PAGE*)	60
LNCaP (IPG-PAGE)	18
PC-3 (IEF-PAGE*)	25
BPH-1 (IEF-PAGE*)	24
Rhabdomyosarcoma (IEF-PAGE*)	29
Normal human fibroblasts (IEF-PAGE*)	38
* modification [20]	

 The same procedure was applied to construct other synthetic maps of proteins from cultured human cell lines, although much fewer 2DE images were used for those maps. 

Thus, each of the constructed maps contained data on the electrophoretic properties of the protein fractions (represented by their coordinates) in the corresponding object. These maps were in *.jpg format (with a resolution of at least 300 dpi), constituting Level 1 of the database. Further studies and analysis of data on proteins were based on the information contained in the Level 1 maps. Therefore, the synthetic maps represented original modules enabling the characterizing and formalizing of the biochemical properties of the proteins studied. There are currently seven modules in PCP (Table 1). There is a special panel enabling navigation among the modules. The 2D maps are scaleable, and the user can mark certain proteins on the maps and create links (buttons) for accessing other levels-second, third, and fourth-that contain data on the proteins. The database also automatically displays 2D coordinates (along the two fractioning axes) as the cursor moves around the map. The general organisation of the PCP database is presented in Fig. 3.

The third step in the proteomic study was to identify individual protein fractions. The proteins were mainly identified by mass-spectrometry: the results are presented in Table 1.

As Table 1 shows, there is a total of 359 identified proteins in PCP. Among them there are many well-known proteins, such as the enzymes responsible for glycolysis (glyceraldehyde-3-phosphate dehydrogenase, triose-phosphate isomerase, etc.) and other metabolic processes, as well as cytoskeletal (actin, transgelins, etc.) and mitochondrial (porins, superoxide dismutase, etc.) proteins. Some of the identified proteins, for instance transgelins [21, 22], were represented with several isoforms.

**Fig. 4:**
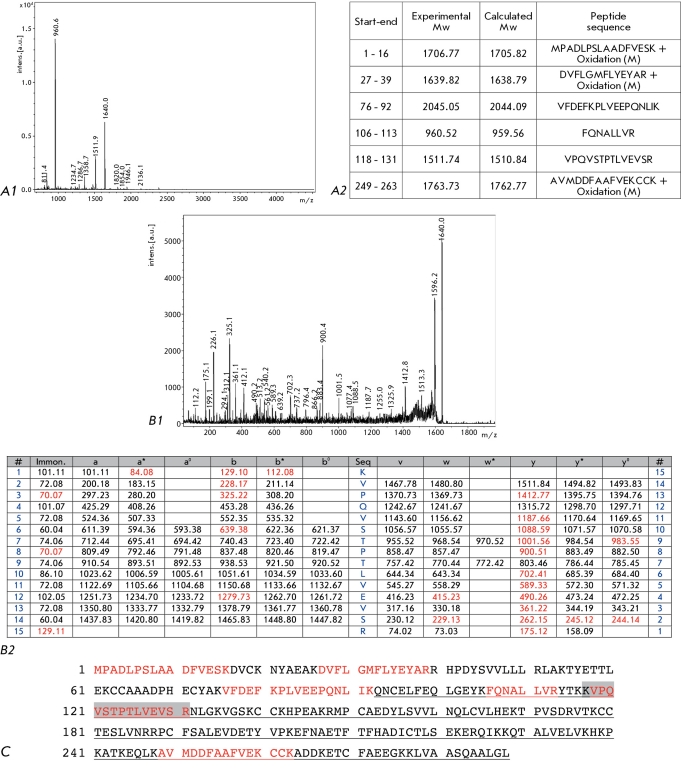
Results of mass-spectrometric identification of the PRO 2675 protein. A. Mass-spectrum of tryptic peptides. (1) MALDI-TOF MS data. (2) Peptide identification by Mascot. B. Mass-spectrum of one of the tryptic peptides (1) MALDI-TOF MS data. (2) Peptide identification by Mascot. C. Amino acid sequence of the PRO 2675 protein (records AAF69644.1 GI:7770217 in the NCBI Protein database); amino acid residues of the tryptic peptides are printed in red; the peptide's whose sequence was identified by MALDI-TOF MS/MS and is highlighted in grey; the sites corresponding to the albumin domain are underlined.

We paid particular attention to the identification of the protein fractions which differed qualitatively or quantitatively in the prostate tissue samples from patients with BHP and PCa. We previously reported on the results of identification of two potential PCa biomarkers, the proteins AGR2 [14] and Dj-1 [26]. In total, we succeeded in identifying 17 potential PCa biomarkers, some of which are new. Table 2 provides a short description of the potential PCa biomarkers. For example, Fig. 4 shows the results of MS identification of one of the new biomarkers, protein PRO2675, which contains an albumin domain in its primary structure.

**Fig. 5:**
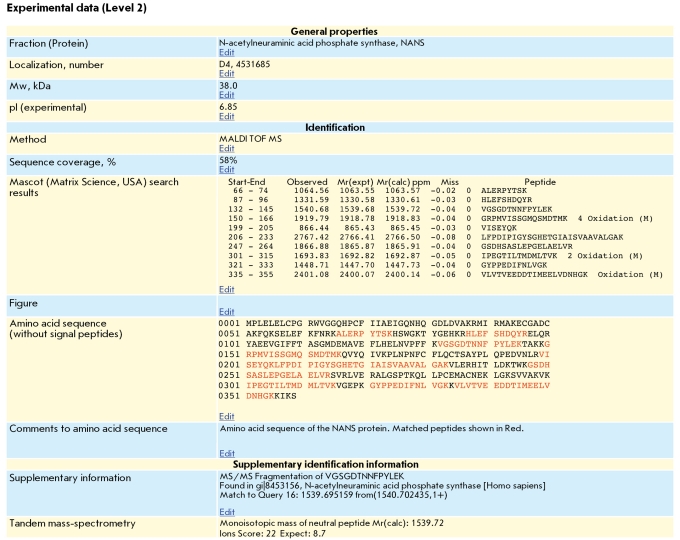
Level 2 fields for the NANS protein.

For each protein identified (and marked with a "button" on the 2D map), the second information Level was formed, comprising a standardized system of 15 fields for the entry of text and graphical data obtained during characterization of the corresponding protein fraction. In four fields, general information about the protein is entered, in the next six fields the identification results are entered, and in the other five fields additional information is entered. The filled Level 2 fields for one of the potential PCa biomarkers, protein NANS (N **-** acetylneuraminic acid phosphate synthase), are shown in Fig. 5.

The same protein could be present in more than one object. Therefore, within Level 2, one can use the control panel to create cross-links between identical proteins in different modules. An example of such cross-referencing for protein Dj-1 is presented in Fig. 6.

**Table 2:** Potential PCa biomarkers in the "Prostate biopsy samples, PCa and BPH" module and other PCP modules

Unique identifier*	Protein (synonyms and symbol in PCP)	Numbers in NCBI** and*Swiss-Prot*	Additional information in PCP and references***
5653580	Ferritin light chain complex (K-(L)F)	182516,*P02792*	[15]
4785508 (4799550)	Chaperonin (HSPD1)	31542947, NP_002147, 118190,*P10809*	Found in rhabdomyosarcoma cells; {Bindukumar B. et al. 2008, 18646040}
4716560 (4756612)	Protein-disulphide isomerase (ER60)	7437388,*P30101*	[15]
4531685	N-acetylneuraminat phosphate synthase (NANS)	12652539, AAH00008, NP_061819, 605202, Q9NR45	Found in rhabdomyosarcoma cells; [15]
4502675	Annexin 2, isophorm 2 (ANXA2-i2)	4757756, NP_004030, 151740,*P07355*	{Shiozawa Y. et al. 2008, 18636554; Hastie C. et al. 2008, 18211896}
4454692	Unknown protein PRO2675 containing the albumin domain (PRO2675)	7770217	[15]
4447605	Protein 29 of endoplasmic reticulum, isophorm 1 (Erp29)	5803013, NP_006808, 602287,*P30040*	{Myung J.K. et al. 2004, 15598346}
4352630 (4342620)	Dj-1 protein (Dj-1)	50513593, 1SOA_A, 606324,*Q99497*	{Bindukumar B. et al. 2008, 18646040}
4356607 (4344615)	Dj-1 protein, electrophoretic isophorm (Dj-1-ei)	31543380	
4336712 (4301795)	Prostatic binding protein (neuropolypeptide h3, PEBP1)	21410340, AAH31102, 604591,*P30086*	[15]; {Li et al. 2008, 18161940; Woods Ignatoski K.M. et al. 2008, 18722266}
4286750 (4290620)	NM23B-protein, nucleoside phosphate kinase B	4505409, NP_002503, 156491,*P22392*	{Johansson B. et al. 2006, 16705742}
4255880	Unnamed protein (NEDO human cDNA sequencing project, tissue type = "testis") (NEDO)	21758704, BAC05360	
4204630	Fatty acid binding protein, isophorm 5 (E-FABP)	30583737, AAP36117, 605168,*Q01469*	[15]; {Morgan E.A. et al. 2008, 18360704}
4279900	AGR2 (AGR2)	37183136, AAQ89368, 606358.*Q4JM47*	[14, 15]; {Zhang J.S. et al. 2005, 15834940; weitzig D.R. et al. 2007, 17694278}
41811130	Hystone H3, 3A family (H3f3a)	55665435	[15]
4161675	Unknown protein PRO2044 containing the albumin domain (PRO2044)	6650826	[15]
4021610	S100 calcium binding protein A11 (S100A11)	12655117, AAH01410, 603114,*P31949*	{Rehman I. et al. 2004, 15668896; Schaefer K.L. et al. 2004, 15150091}

* Numbers in the "LNCaP (IEF-2DE Modification)" module are given in parenthesis

** Numbers from the NCBI databases are given in the following order: Protein, Genbank and/or Nucleotide, OMIM

*** Publications listed in the References section of this article are given in square brackets; publications from the PubMed database are given in curly brackets.

Note: a new potential biomarkers are shown in bold

**Fig. 7:**
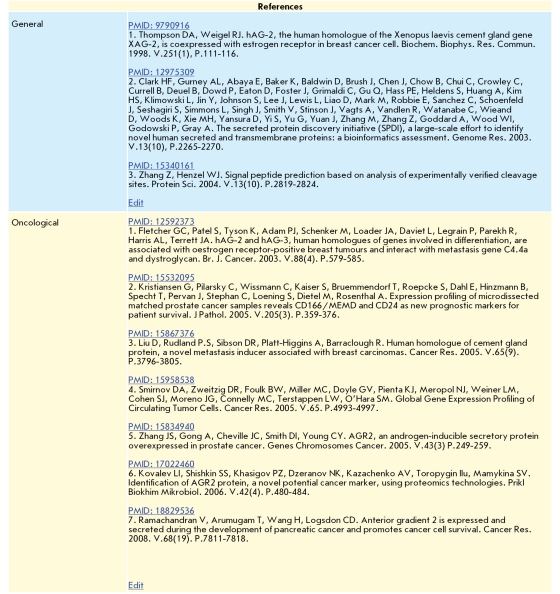
Level 3 fields for specially selected references to publications on the AGR2 protein.

The majority of the 359 identified fractions were well-known proteins (and/or their electrophoretic isophorms) described in the literature and various databases. Some information on those proteins, relating to the PCP scope, constituted the third information Level of the database. Level 3 is a standardized system of 23 fields for the entry of text and graphical data. In twelve fields information about the protein is entered, in the next six fields information about the gene coding for the protein is entered, and in the next three fields information about the protein's polymorphism is entered. In the other fields, selected references to publications about the general properties of the proteins, as well as its oncological properties, are entered (Fig. 7).

 The Level 3 text fields can contain hyperlinks to various Internet databases, such as NCBI's Protein, OMIM and PubMed, and SwissProt. This feature allowed the creation of the fourth information Level, providing the user with prompt access to international databases containing, in particular, the results of human genome sequencing. 

**Fig. 6:** Control panel with cross-references for the Dj-1 protein from the "Prostate biopsy samples, PCa and BPH" module.

Identical proteins in other modules				
Module	Point	Link	Disconnect
LNCaP (IPG-2DE)	4301630.Dj-1	Link	Disconnect
LNCaP (IEF-2DE modification)	4342635.Dj-1	Link	Disconnect
Rhabdomyosarcoma	4342690.DJ-1	Link	Disconnect
PC3	4342630.DJ-1	Link	Disconnect
Add

 The PCP database is an interactive MySQL-based web resource located at http://ef.inbi.ras.ru and can be accessed from any computer connected to the Internet using the Mozilla Firefox and Microsoft Internet Explorer browsers. There are three access permission categories: "Guest," "Manager," and "Administrator," each giving certain rights for working with PCP. In particular, users with "Manager" access permission can make entries into and correct the Level 2 and 3 fields, while users with the "Administrator" category access permission, have the ability to expand the database by creating new modules and new functional elements. Users with "Guest" access can browse all fields but cannot edit them. 

 In conclusion, our work resulted in the creation of an original multi-module national database entitled "Prostate Cancer Proteomics," which summarizes data on the proteins in prostate tissue collected from patients with BHP and PCa, as well as on proteins in several human cell lines. This is very promising in the further use of proteomic and other biochemical data. We are hopeful that the PCP database will be useful to biochemists and other biomedical scientists, making their research on PCa more efficient. 

## Abbreviations

2DE: two-dimensional gel electrophoresis
BPH: benign prostate hyperplasia
PCa: prostate cancer
PCP: prostate cancer proteomics

## References

[1] Gottlieb B., Beitel L.K., Wu J.H., Trifiro M. (2004). Hum. Mutat..

[2] Westbrook J.A., Wheeler J.X., Wait R., Welson S.Y., Dunn M.J. (2006). Electrophoresis..

[3] Kandasamy K., Keerthikumar S., Goel R., Mathivanan S., Patankar N., Shafreen B., Renuse S., Pawar H., Ramachandra Y.L., Acharya P.K., Ranganathan P., Chaerkady R., Keshava Prasad T.S., Pandey A. (2009). Nucleic Acids Res..

[4] Vizcaino J.A., Cote R., Reisinger F., Barsnes H., Foster J.M., Rameseder J., Hermjakob H., Martens L. (2010). Nucleic Acids Res..

[5] Stamey T.A., Caldwell M., McNeal J.E., Nolley R., Hemenez M., Downs J. (2004). J. Urol..

[6] Zhang J.S., Gong A., Cheville J.C., Smith D.I., Young C.Y. (2005). Genes Chromosomes Cancer..

[7] Lim L.S., Sherin K. (2008). Am. J. Prev. Med..

[8] Leman E.S., Getzenberg R.H. (2009). J. Cell Biochem..

[9] Chissov V.I., Starinsky V.V., Petrova G.V. (2008). Zlokachestvennie novoobrazovania v Rossii v 2008. Zabolevaemost i smertnost (Malignant Tumors in Russia in 2008. Morbidity and Mortality).

[10] Blokhin N.N. (2008). Zlokachestvennie novoobrazovania v Rossii i SNG v 2002 (Malignant Tumors in Russia and CIS in 2002).

[11] Jemal A., Siegel R., Ward E., Murray T., Xu J., Smigal C., Thun M.J. (2007). CA Cancer J. Clin..

[12] Maddams J., Brewster D., Gavin A., Steward J., Elliott J., Muller H. (2009). Br. J. Cancer..

[13] Primrose S.B., Twyman R.M. (2008). Genomika. Rol v Medicine (Genomics. Applications in Human Biology)..

[14] Kovalyov L.I., Shishkin S.S., Hasigov P.Z., Dzeranov N.K., Kazachenko A.V., Kovalyova M.A., Toropygin I.Y., Mamikina S.V. (2006). Prikladnaya biokhimia i mikrobiologia (Applied Biochemistry and Microbiology)..

[15] Shishkin S.S., Dzeranov N.K., Totrov K.I., Kazachenko A.V., Eremina L.S., Kovalyova M.A., Toropygin I.Y. (2009). Urologiia (Urology)..

[16] Kogan M.I., Loran O.B., Petrov S.B. (2006). Radikalnaya khirurgia raka predstatelnoy zhelezi (Radical Surgery of Prostate Cancer).

[17] Shishkin S.S., Kovalyov L.I., Kovalyova M.A., Krakhmaleva I.N., Eremina L.S., Makarov  A.A., Lisitskaya K.V., Loran O.B., Veliev E.I., Okhrits V.E. (2009). Problemi ranney diagnostiki raka prostati i vosmozhnosti primenenia novih potencialnih biomarkerov (The Problems of Early Diagnostics of Prostate Cancer and Possibilities of Using of New Potential Biomarkers.).

[18] Chernikov V.G., Terehov S.M., Krohina T.B., Shishkin S.S., Smirnova T.D., Lunga I.N., Adnoral N.V., Rebrov L.B., Denisov-Nikolsky Y.I., Bikov V.A. (2001). Bull. eksp. biol. med. (Bulletin of Experimental Biology and Medicine)..

[19] Krohina T.B., Shishkin S.S., Raevskaya G.B., Ershova E.S., Mirochnik V.V., Bubnova E.N., Kucharenko V.I. (1996). Bull. eksp. biol. med. (Bulletin of Experimental Biology and Medicine)..

[20] Kovalyov L.I., Shishkin S.S., Efimochkin A.S., Kovalyova M.A., Ershova E.S., Egorov T.A., Musalyamov A.K. (1995). Electrophoresis..

[21] Eremina L.S., Kovalyov L.I., Shishkin S.S., Toropygin I.Y., Burakova M.I., Kovalyova M.A., Makarov A.A., Dzeranov N.K., Kazachenko A.V., Totrov K.I., Kononkov I.V., Loran O.B. (2007). Vopr. biol. med. farm. khimii (Problems of Biological, Medical, and Pharmaceutical Chemistry)..

[22] Kovalyova M.A., Kovalyov L.I., Eremina L.S., Makarov A.A., Burakova M.I., Toropygin I.Y., Serebryakova M.V., Shishkin S.S., Archakov A.I. (2008). Biomedicinskaya khimia (Biomedical Chemestry)..

[23] Anderson N.G., Anderson L. (1996). Electrophoresis..

[24] Shishkin S.S., Kovalyov L.I., Gromov P.S. (2000). Mnogolikost sovremennoy genetiki cheloveka (The Variety of Contemporary Human Genetics).

[25] Cumings D. (1982). Clin. Chem..

[26] Loran O.B., Veliev E.I., Okhrizts V.E., Lisitskaya K.V., Eremina L.S., Kovalyov L.I., Kovalyova M.A., Shishkin S.S. (2010). Eur. Urol. Suppl..

